# Transcriptomic alterations in cortical astrocytes following the development of post-traumatic epilepsy

**DOI:** 10.1038/s41598-024-58904-z

**Published:** 2024-04-10

**Authors:** John Leonard, Xiaoran Wei, Jack Browning, Erwin Kristobal Gudenschwager-Basso, Jiangtao Li, Elizabeth A. Harris, Michelle L. Olsen, Michelle H. Theus

**Affiliations:** 1 Department of Biomedical Sciences and Pathobiology, Faculty of Health Sciences, Virginia Tech, 970 Washington Street SW, Life Sciences I; Rm 249 (MC0910), Blacksburg, VA 24061 USA; 2 School of Neuroscience, Virginia Tech, Blacksburg, VA 24061 USA

**Keywords:** Seizures, Traumatic brain injury, Cst3, Gliosis, GFAP, Neuropeptide amidating enzyme, Pam, Ccl4, Molecular biology, Neuroscience

## Abstract

Post-traumatic epilepsy (PTE) stands as one of the numerous debilitating consequences that follow traumatic brain injury (TBI). Despite its impact on many individuals, the current landscape offers only a limited array of reliable treatment options, and our understanding of the underlying mechanisms and susceptibility factors remains incomplete. Among the potential contributors to epileptogenesis, astrocytes, a type of glial cell, have garnered substantial attention as they are believed to promote hyperexcitability and the development of seizures in the brain following TBI. The current study evaluated the transcriptomic changes in cortical astrocytes derived from animals that developed seizures as a result of severe focal TBI. Using RNA-Seq and ingenuity pathway analysis (IPA), we unveil a distinct gene expression profile in astrocytes, including alterations in genes supporting inflammation, early response modifiers, and neuropeptide-amidating enzymes. The findings underscore the complex molecular dynamics in astrocytes during PTE development, offering insights into therapeutic targets and avenues for further exploration.

## Introduction

Traumatic brain injury (TBI) stands as one of the leading causes of disability and death worldwide, resulting from various accidents, sports-related injuries, and military combat situations^1^. It is a complex event, encompassing primary and secondary injury mechanisms that inflict significant damage to neural tissue. The primary injury occurs immediately upon impact and leads to direct structural damage, while the secondary injury involves a series of biochemical and cellular processes that evolve over time^2^. Beyond the immediate consequences, TBI can trigger long-term neurological complications, including post-traumatic epilepsy (PTE), a recurring seizure disorder following brain injury after trauma^3,4^. PTE affects a substantial proportion of individuals who have experienced a severe brain injury, with post-TBI incidences ranging from 2 to 50%, depending on injury severity, age, and comorbidities^5,6^, highlighting the critical need for a deeper understanding of its underlying mechanisms, however, the brain's complexity and diverse interactions of genetic and environmental factors have long perplexed researchers seeking to comprehend the cascade of events leading to PTE.

Astrocytes, a type of glial cell in the brain, play a critical role in maintaining the brain's health and functionality. These star-shaped cells are involved in various functions such as providing structural support to neurons, regulating neurotransmitter levels, and maintaining the blood–brain barrier^7^. In response to injury, astrocytes release pro-inflammatory molecules and may contribute to the development of an epileptogenic environment by altering synaptic function, promoting neuronal excitability, and influencing the formation of epileptic networks^8–10^. Understanding the role of astrocytes in the context of PTE is a key avenue of research, potentially leading to new strategies for diagnosing, preventing, or treating this challenging condition. Astrocyte transcriptomics in particular could be regarded as an important frontier for understanding and treating PTE.

The present investigation explored the genome-wide mRNA transcript changes using bulk sequencing in cortical astrocytes associated with PTE. Understanding alterations in astrocyte transcriptomics may advance our understanding of epilepsy's development by shedding light on the intricate molecular processes regulating glial function, offering insights into potential targets for therapeutic interventions and more personalized treatment strategies.

## Results

### Transcriptomic signature of astrocytes isolated from the cortex of sham, PTE+ and PTE− mice

**Figure 1:**
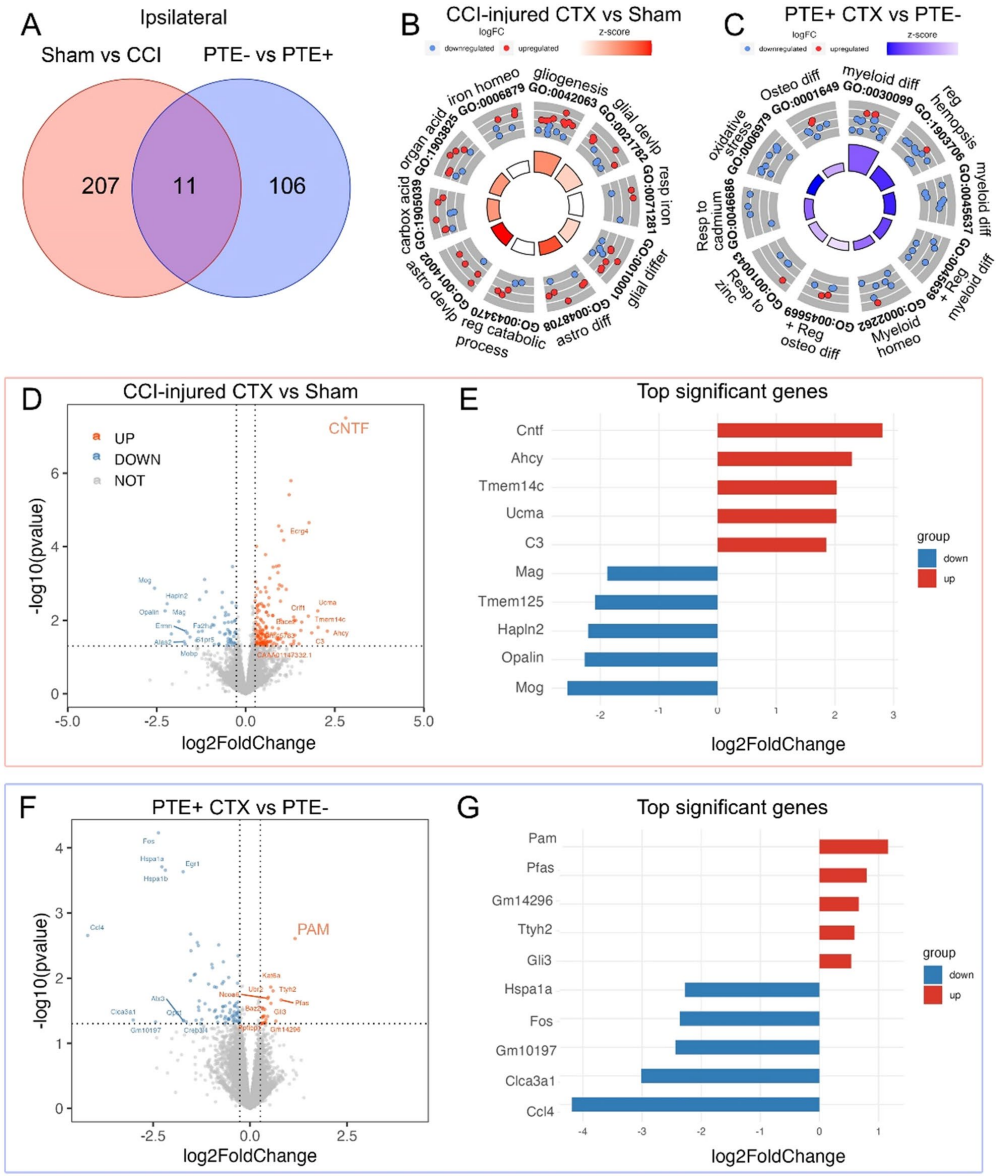
Transcriptomic analysis of ipsilateral cortical astrocytes. (**A**) Venn diagram of DEGs between sham and CCI-injured mice, as well as PTE+ and PTE− mice in the ipsilateral cortex at 4-months post-injury. (**B**) GO circle plot showing the top ten gene ontology enrichment terms in sham versus CCI mice. (**C**) GO circle plot showing the top ten gene ontology enrichment terms in PTE+ versus PTE− mice. (**D**) Volcano plot of DEGs between sham and CCI-injured mice. (**E**) Bar graph of the top five upregulated and downregulated genes between sham and CCI-injured mice. (**F**) Volcano plot of DEGs between PTE+ and PTE− mice post-CCI. (**G**) Bar graph of the top five upregulated and downregulated genes between PTE+ and PTE− mice.

Dysregulation of astrocytes has been implicated in the development and progression of epilepsy^11,12^. Understanding how the transcriptomic landscape is altered in astrocytes will uncover key molecular pathways and reveal potential therapeutic targets to prevent the development of PTE. We assessed transcriptomic changes by performing RNA-Seq on astrocytes isolated from the ipsilateral and contralateral cortex of sham (n = 5) and severe CCI-injured (n = 15) mice at 4 months post-injury. Mice were stratified based on seizure development (PTE+ vs. PTE−), as previously reported^13^. In total, among the severe CCI-injured mice, five mice were PTE+ (33%) and ten mice were PTE− (67%). None of the sham mice exhibited PTE. Transcriptomic analysis was performed in conjunction with gene ontology pathway enrichment to detect differentially expressed genes (DEGs) and biological processes (BP) respectively. Astrocytes isolated from the cortex were verified based on expression of *Gfap, Slc1a3* and lack of *Itgam*, *Rbfox3* and *Mbp* (Supplemental Fig. 1). Ipsilateral cortical astrocytes showed 207 DEGs that were altered in CCI-injury compared to sham and 106 DEGs altered in CCI-injured PTE+ compared to PTE− mice, while 11 genes were common between the comparison groups (Fig. 1A). The top five (Log2FC) upregulated genes in sham vs. CCI were *Cntf*, *Ahcy*, *Tmem14c*, *Ucma*, and *C3*, while the top five (Log2FC) downregulated genes were *Mag*, *Tmem125*, *Hapln2*, *Opalin*, and *Mog* (Fig. 1D,E). When comparing PTE+ versus PTE− genes, we found that the top five upregulated genes (Log2FC) were *Pam*, *Pfas*, *Gm14296*, *Ttyh2*, and *Gli3*, while the top five downregulated genes (Log2FC) were *Hspa1a*, *Fos*, *Gm10197*, *Clca3a1*, and *Ccl4* (Fig. 1F,G). Gene ontology of the ipsilateral injured cortex vs. sham revealed changes in genes associated with gliogenesis, glial development, and differentiation (Fig. 1B), while myeloid cell differentiation and homeostasis are predominant biological processes associated with gene changes in PTE+ mice (Fig. 1C). These data highlight key astrocytic genes altered in the ipsilateral cortex of PTE+ mice including upregulation of peptidylglycine α-amidating monooxygenase (*PAM*), an essential enzyme for the synthesis of amidated neuropeptides. Notably, none of these top upregulated or downregulated genes are astrocyte-restricted but play key roles in astrocyte function.

**Figure 2:**
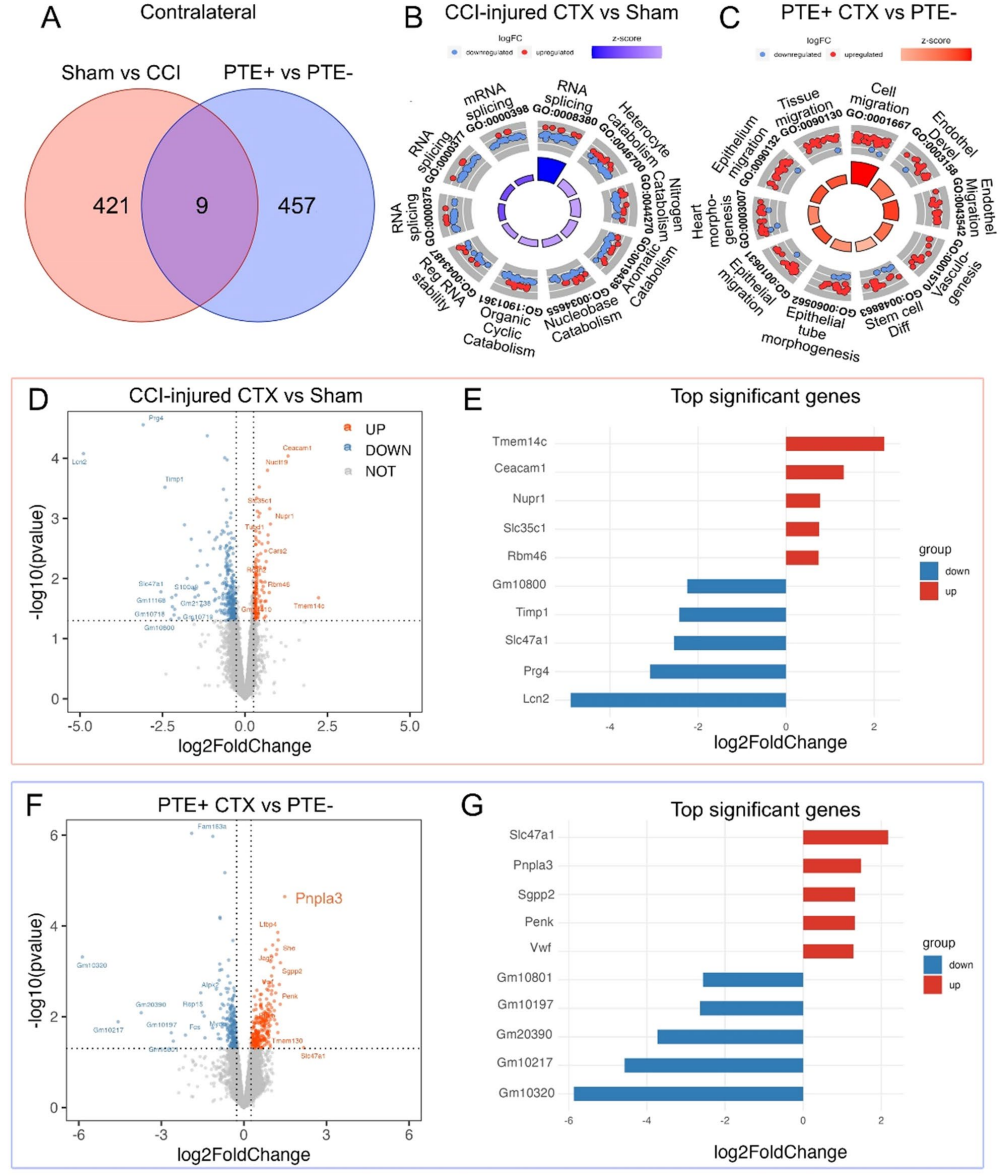
Transcriptomic analysis of contralateral cortical astrocytes. (**A**) Venn diagram of DEGs between sham and CCI-injured mice, as well as PTE+ and PTE− mice in the contralateral cortex at 4 months post-injury. (**B**) GO circle plot showing the top ten gene ontology enrichment terms in sham versus CCI mice. (**C**) GO circle plot showing the top ten gene ontology enrichment terms in PTE+ versus PTE− mice. (**D**) Volcano plot of DEGs between sham and CCI-injured mice. (**E**) Bar graph of the top five upregulated and downregulated genes between sham and CCI-injured mice. (**F**) Volcano plot of DEGs between PTE+ and PTE− mice post-CCI. (**G**) Bar graph of the top five upregulated and downregulated genes between PTE+ and PTE− mice.

Evidence suggests an important functional and structural basis of contralateral interictal activity in focal, unilateral epilepsy^14^. We next addressed associated changes in the contralateral cortical astrocytic transcriptome in our focal CCI model^13^. CCI injury showed changes in 421 genes compared to sham, and when comparing PTE+ versus PTE− injured-mice we observed 457 genes that were altered in astrocytes from animals that developed generalized seizures (Fig. 2A). Only nine genes were commonly expressed between these groups. The top five (Log2FC) upregulated genes between sham and CCI were *Tmem14c*, *Ceacam1*, *Nupr1*, *Slc35c1*, and *Rbm46*, and the top five (Log2FC) downregulated genes were *Gm10800*, *Timp1*, *Slc47a1*, *Prg4*, and *Lcn2* (Fig. 2D,E). The top five (Log2FC) upregulated genes in PTE+ astrocytes compared to PTE− were *Slc47a1*, *Pnpla3*, *Sgpp2*, *Penk*, and *Vwf*, while the top five (Log2FC) downregulated genes were annotated genes without a canonical name including *Gm10801*, *Gm10197*, *Gm20390*, *Gm10217*, and *Gm10320* (Fig. 2F,G). Gene ontology of the post-injury contralateral cortex versus sham predominantly showed top GO terms associated with RNA splicing and stability as well as catabolic processes (Fig. 2B), while GO terms in PTE+ mice related to ameboidal-type cell migration, vasculogenesis, and endothelial processes (Fig. 2C). Interestingly, *Tmem14c*, a key player in mitochondrial heme metabolism^15^, was a top gene upregulated in both ipsilateral and contralateral astrocytes in CCI-injured compared to sham mice. These findings show significant changes in astrocytic genes in the uninjured contralateral cortex of PTE+ mice that are distinct from the ipsilateral cortex.

**Figure 3:**
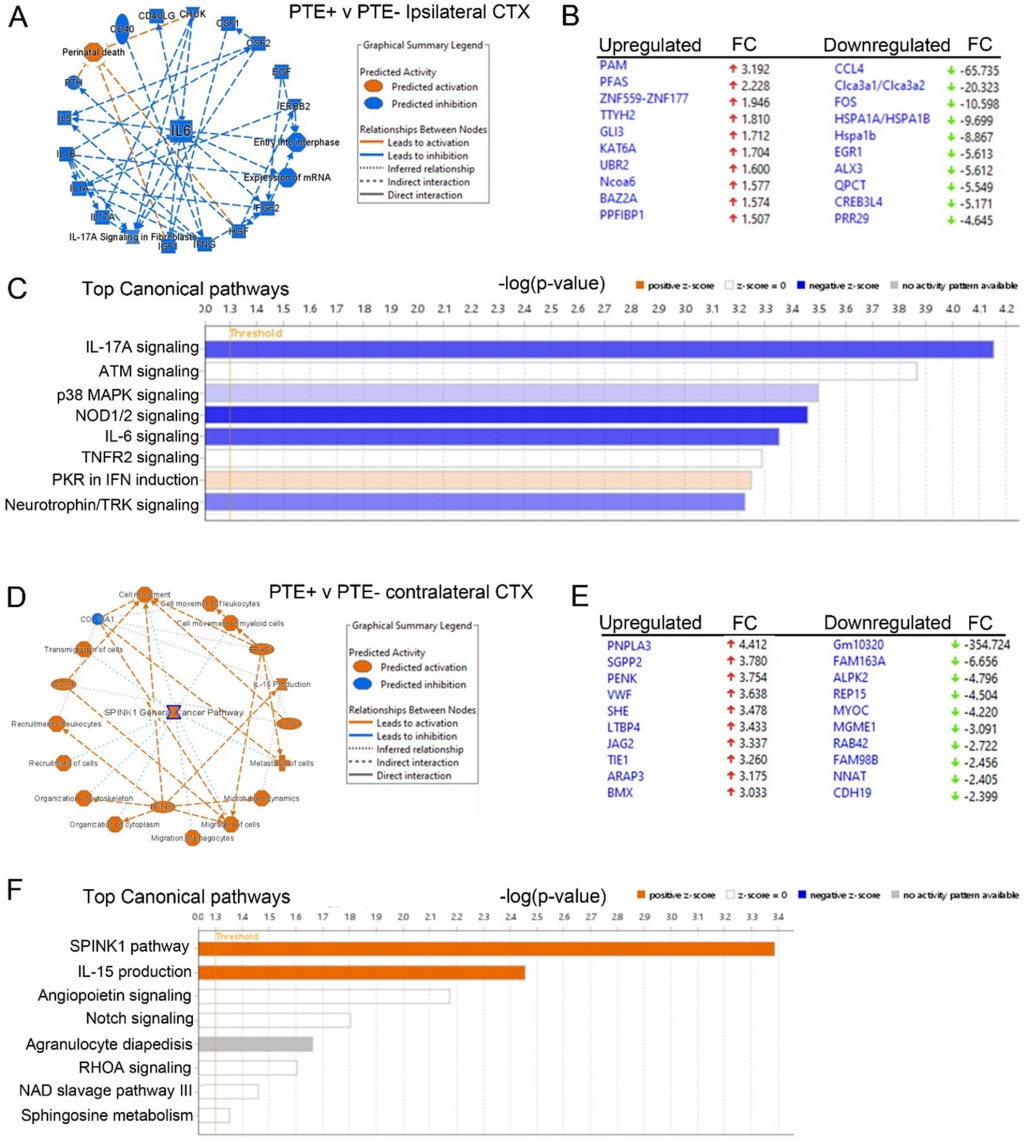
Ingenuity pathway analysis using RNA-seq data from ipsilateral and contralateral cortical astrocytes of PTE+ and PTE− mice. (**A**) Graphical summary showing predicted inhibition of Il6 in PTE+ mice and other pathways with inferred relationship to Il6 including IFNG, IGF1, IL1A, IL5, IL17A. (**B**) Top ready analysis showing the top upregulated and top downregulated genes based on fold-change. (**C**) The top 8 canonical pathways based on + or − z-score. (**D**) Graphical summary showing predicted activation of serine peptidase inhibitor Kazal type 1 (SPINK1) general cancer pathway, as well as IL-15 production, KLF4, SOX4 and migration of phagocytes/myeloid cells. (**E**) Top ready analysis showing the top upregulated and top downregulated genes based on fold-change. (**F**) The top 8 canonical pathways based on + or—z-score.

Ingenuity pathway analysis (IPA) was performed to comprehensively analyze differential gene expression and infer predicted downstream effects for potential mechanistic targets. A graphical summary of the major canonical pathways, upstream regulators, and biological processes shows the significant genes altered in PTE+ cortical astrocytes reveal predicted inhibition of Il6 and other pathways with inferred relationship to Il6 including IFNG, IGF1, IL1A, IL5, IL17A (Fig. 3A) based on top ready analysis (Fig. 3B) and other significantly altered genes. The top 8 canonical pathways show a negative z-score for Il17A, p38 MAPK, NOD1/2, IL-6 and a positive z-score for PKR in interferon (IFN) signaling (Fig. 3C). The IPA summary of contralateral PTE+ cortical astrocytes shows predicted activation of serine peptidase inhibitor Kazal type 1 (SPINK1) general cancer pathway, as well as IL-15 production, KLF4, SOX4 and migration of phagocytes/myeloid cells (Fig. 3D). Based on top ready analysis genes (Fig. 3E) and other statistically significant genes, top canonical pathways include SPINK1, IL-15 production with positive z-scores (Fig. 3F).

**Figure 4:**
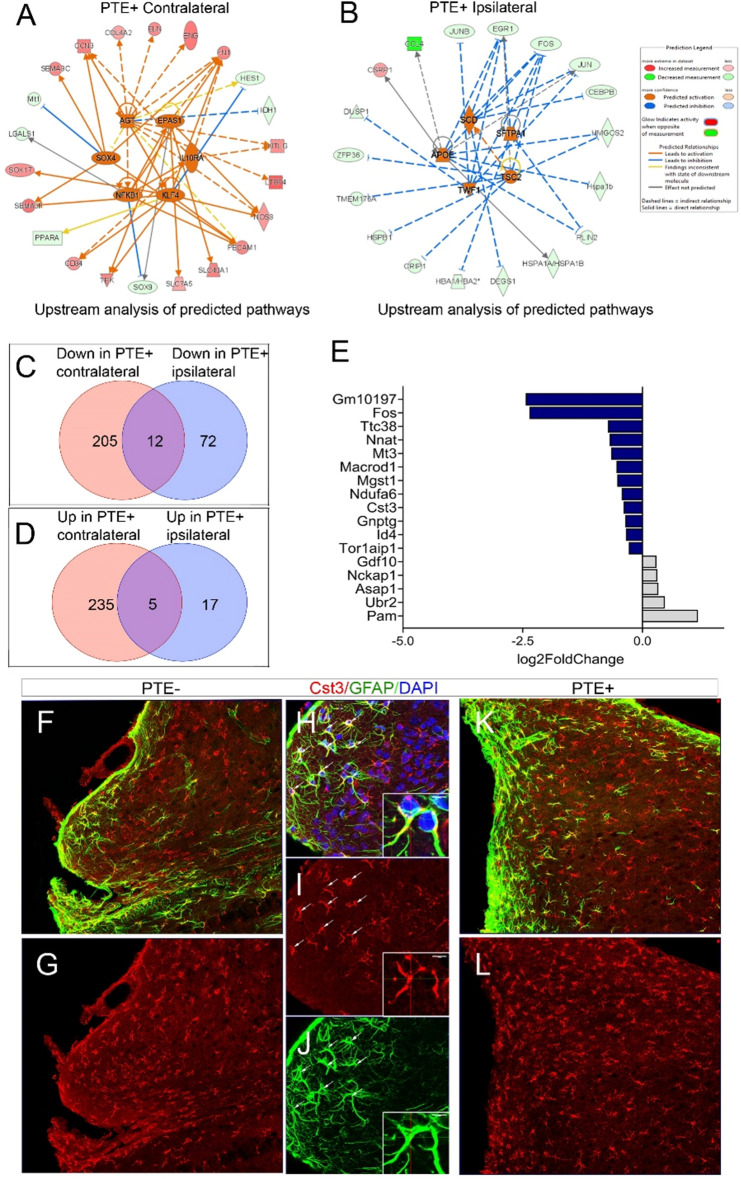
Synopsis of IPA and DEGs in PTE+ cortical astrocytes from both ipsilateral and contralateral cortex. (**A**) Graphical IPA summary of contralateral PTE+ cortical astrocytes showed downregulation of genes such as MT1, SOX9, and PPARA, with upregulation of several genes including SOX17, and CD34 among others via predicted activation of AGT, EPAS1, SOX4, NFKB1, KLF4, and IL10RA (**B**) Graphical IPA summary of ipsilateral PTE+ cortical astrocytes demonstrated downregulation of CCL4 via predicted activation of APOE, and other genes such as FOS were downregulated via predicted activation of SCD, SFTPA1, TSC2, and TWF1. (**C**) Venn diagram of the number of downregulated genes between contralateral and ipsilateral cortical astrocytes in PTE+ mice. (**D**) Venn diagram of the number of upregulated genes between contralateral and ipsilateral cortical astrocytes in PTE+ mice. (**E**) Bar graph of the 17 DEGs found across both hemispheres based on log2FoldChange. (**F**-**J**) Representative max z-projection of GFAP and Cst3 immunohistochemistry in the PTE− cortex (**K**-**L**) and PTE+ cortex 4 months post-CCI injury. Cst3 expression is present on GFAP-positive astrocytes, star-shaped microglia in the peri-lesion area.

A broader IPA synopsis of PTE+ cortical astrocytes yielded predicted activation of several pathways. Analysis of contralateral PTE+ cortical astrocytes showed downregulation of genes such as MT1, SOX9, and PPARA, with upregulation of several genes including SOX17, and CD34 among others via predicted activation of AGT, EPAS1, SOX4, NFKB1, KLF4, and IL10RA (Fig. 4A). Analysis of ipsilateral PTE+ cortical astrocytes demonstrated downregulation of *Ccl4* via predicted activation of APOE, and other genes such as *Fos* were downregulated via predicted activation of SCD, SFTPA1, TSC2, and TWF1 (Fig. 4B). Overall, we found that 217 genes were downregulated in contralateral PTE+ cortical astrocytes, 84 genes were downregulated in ipsilateral PTE+ cortical astrocytes, and 12 genes were downregulated in both contralateral and ipsilateral samples (Fig. 4C). In contrast, 240 genes were upregulated in contralateral PTE+ cortical astrocytes, 22 genes were upregulated in ipsilateral PTE+ cortical astrocytes, and 5 genes were upregulated in both contralateral and ipsilateral samples (Fig. 4D). In total, 17 genes were differentially expressed in both contralateral and ipsilateral samples of PTE+ cortical astrocytes. These included downregulation of *GM10197, FOS, TTC38, NNAT, MT3, MACROD1, MGST1, NDUFA6, CST3, GNPTG, ID4,* and *TOR1AIP1*, and upregulation of *GDF10, NCKAP1, ASAP1, UBR2,* and *PAM* (Fig. 4E). *Pam* was the most upregulated gene, and the most downregulated genes were *GM10197*, and *Fos* based on log2FoldChange in both hemispheres.

Finally, we previously identified Cystatin 3 (*Cst3*)*,* a highly expressed gene in astrocytes^16^, to be altered in the ipsilateral and contralateral hippocampus of PTE+ astrocytes via bulk RNA-seq of hippocampal astrocytes from PTE+ mice and further confirmed by immunohistochemistry ^13^. We demonstrate that this is the only gene influenced across all brain regions in isolated astrocytes from PTE+ mice. Immunohistochemistry of Cst3 on GFAP-expressing peri-lesional cortical astrocytes is evident at 4 months post-CCI injury in both PTE− and PTE+ mice (Fig. 4 F-L), however, we observed its expression was more prominent on IBA1-positive microglia. The cell-type specific role of Cst3 may be a viable target for further exploration.

## Discussion

There is a growing recognition that astrocytes play a significant role in epileptogenesis^17^. Neuronal changes, such as those induced by TBI, lead to reactive astrogliosis, wherein gene expression and morphological alteration are prominent^18,19^. Studies using human brain tissues have found that epilepsy is associated with astrocytic and microglial activation^20^. Moreover, astrocyte cytokines play a pivotal role in the development of seizures, influencing the inflammatory milieu within the brain. Their release can contribute to a cascade of events that enhance seizure susceptibility and potentially exacerbate neuronal damage associated with epileptic activity. The current study reveals that astrocytes from the CCI-injured cortex display key changes in transcripts, including upregulation of *Cntf, Ahcy, Tmem14c, Ucma,* and *C3* at months post-injury, whose biological processes relate to gliogenesis, glial development and differentiation, as well as iron homeostasis and respiration. This correlates with a typical pattern of gliosis seen in the progressive stages of TBI^21–23^. However, the ipsilateral CCI-injured mice that developed PTE, uniquely show upregulation of several top genes, including *Pam, Pfas, Ttyh2* and *Gli3* and reduced levels of *Fos, Hspa1a, Clca3a1* and *Ccl4*. Interestingly, this transcriptomic shift correlates with biological processes related to myeloid cell homeostasis and differentiation, hematopoiesis, response to zinc ion, which inhibits synaptic GABA-A receptors^24^, as well as oxidative stress. This suggests astrocyte activity may overlap with features of myeloid cell functions such as antigen presentation, phagocytosis, and inflammation in mice that develop seizures.

Numerous clinical and animal investigations have elucidated the intricate interplay between seizures and the cytokine-mediated inflammatory response^25^. Investigations centered on molecular markers of neuroinflammation hold significant importance and may contribute to a deeper understanding of epileptogenesis in TBI. Altered gene expression in the ipsilateral cortical astrocytes of PTE+ mice support inflammation as the top canonical pathways including Il17A, NOD1/2, IL-6, TNFR2 and PKR interferon (IFN) signaling. Patients with epilepsy exhibit higher IL-17 serum and CSF levels, and this phenotype is associated with seizure severity^26^, as well as increased IL-6^27^, and TNFR2, whose global loss is associated with acute seizures^28^. Anomalies in cytokine expression and immune cell activity have been noted in both seizure-afflicted patients and animal models of seizures^27^.

Among the DEGs significantly altered in both hemispheres of PTE+ mice were *Pam, Ubr2, Asap1, Nckap1, Gdf10*. Peptidylglycine α-amidating monooxygenase, or Pam*,* a transmembrane vesicular cuproenzyme and regulator of secretory pathway, is essential for the synthesis of all amidated neuropeptides^29^, which are key players in modulating excitatory/inhibitory balance^30^. Pam has previously been found to be expressed in astrocytes^31,32^ and is transiently increased in the hippocampus in kainic acid (KA) seizures in rats^33^, however, it has not been identified in TBI epileptogenesis. Interestingly, it is involved in the production of amidated neuropeptide Y, which has been shown to exert a significant inhibitory effect on epileptiform activity in the human hippocampal dentate gyrus^34^. It is plausible that increased *Pam* expression in astrocytes may be the result of a demand for amidated peptides, needed to regulate astrocytic functions such as proliferation^35^ or to counteract seizure development.

The mechanism of action for *Ubr2* is not currently understood, but it is a gene involved in regulating the activation of the Wnt/β-catenin pathway, and is involved in the regulation of cell death^36^. While *Ubr2* has not been directly implicated in PTE, it has been associated with multiple sclerosis^37^, and other members of the *Ubr* family have been discussed in the context of neurodevelopmental-related epilepsy^38^. Furthermore, Wnt signaling plays a role in epileptogenesis in the hippocampus^39^. *Nckap1*, a gene involved in neuronal differentiation, interacts directly with *Cyfip2*, which has been implicated in seizures^40,41^. While *Gdf10* has not been a target for epileptogenesis, it may play a role in recovery from TBI as it is involved in axon sprouting after stroke injury^42^. Overall, this pattern of gene expression is consistent with the development of PTE in mice subjected to focal cortical impact. Further studies are needed to experimentally determine the expression and causal relationship of these proteins in PTE.

The downregulation and predicted pathway inhibition of *Ccl4* is notable. Previous studies in rats have found that *Ccl4* gene and protein expression are acutely increased in epilepsy, including in astrocytes^43–45^. *Ccl4* has also been linked to epilepsy in humans^46^. Chemokine CC motif ligand 4 (CCL4) functions as a chemoattractant for many types of immune cells, including macrophages, and also induces calcium mobilization among monocytes and other immune cells^47^. Given the time course dependency of TBI, brain tissue collection at 4 months may constitute a change in the neuroinflammatory status, as Ingenuity Pathway Analysis inferred that type-1 interferon is altered at this chronic time point in the ipsilateral cortex in PTE (not shown), which would serve to suppress *Ccl4*^48^.

Importantly, a number of early response genes, *Egr1, Fos, Jun, Cebpb, JunB,* were all downregulated in the ipsilateral cortical PTE+ astrocytes and are common targets of APOE, SCD, SFTPA1, TWF1, TSC2, which are predicted to be activated. Early response genes (ERGs) are normally minimally expressed, but expression quickly increases after insult or intense neuronal activation^49^. ERG expression in astrocytes has been associated with neuronal survival and neurite outgrowth^50^, and recent findings highlight a novel subpopulation of immediate-early astrocytes (ieAstrocytes)^51^, however, their direct role in epileptogenesis has not been investigated. Fos is upregulated in human epileptic tissue^52^ and its expression in astrocytes has been directly linked with interferon-γ or inflammatory responses, cell proliferation^53^, plasticity, and astrogliosis^54^. In models of epilepsy, ERGs, including Fos, have been correlated with epileptogenesis^55^. The altered expression pattern of ERGs in astrocytes, in addition to changes in inflammatory and neuropeptide-associated genes suggests that PTE+ astrocytes may have shifted their differential gene expression pattern or subpopulation in response to TBI-induced epileptogenesis in an attempt to combat seizure activity.

Lastly, the contralateral cortex yielded upregulation of *Slc47a1* in PTE+ astrocytes compared to PTE−. *Slc47a1* is a gene which encodes the MATE1 protein, a toxin extrusion transporter^56^. Increased expression here may be related to maintaining CNS homeostasis and BBB interactions^57^. Furthermore, activation of the *Spink1* general cancer pathway, as indicated by IPA analysis, is notable, as *Spink1* increases cell proliferation via *Pi3k* and *Akt*^58^. *Pi3k* and *Akt* have been previously noted for their involvement in both TBI and seizures^59,60^. IL-15, whose production was also activated, has not yet been studied in the context of PTE. A pro-inflammatory cytokine whose involvement spans homeostasis and immune response intensity, IL-15 has been studied in stroke models, where it has been found to facilitate crosstalk between astrocytes and microglia, and astrocytic IL-15 in particular potentially causes exacerbated tissue damage and gliosis^61,62^. IL-15 has also recently emerged as a biomarker for prognosis after TBI^63^.

This work presents several potential targets for future studies related to the vital role that astrocytes play in the progression of epileptogenesis following TBI. While bulk and single-cell RNA sequencing of genes involved in TBI has been an intense recent focus^64–66^, the current study reveals novel changes in cortical astrocytes that may be useful for future comparative studies or therapeutic discovery. Further work is needed to determine the interplay between region-specific astrocytes and single-cell analysis of astrocyte subtypes in the development of recurrent generalized seizures in the brain as a consequence of trauma.

## Methods

### Animals

All mice were CD1 males purchased from Charles River. Mice were housed in an AAALAC-accredited facility under 12 h:12 h light/dark cycle, with food and water ad libitum. For EEG/video recording, animals were house individually in 12.5″ × 12.5″ × 15.5″ polycarbonate cages (AAA Plastic Products, Birmingham, AL, USA) with corncob bedding and nesting material. All experiments were conducted in accordance with the NIH Guide for the Care and Use of Laboratory Animals, and with the approval of the Virginia Tech Institutional Animal Care and Use Committee (IACUC; #17-138). Animal work was conducted in accordance with ARRIVE guidelines.

### Controlled Cortical Impact (CCI)

CCI was performed as described previously^67,68^. Succinctly, anesthesia and analgesia were administered before surgery via subcutaneous injection of ketamine (100 mg/kg), xylazine (10 mg/kg), and buprenorphine SR (0.5 mg/kg). Hair on the scalp was removed, and the mice were securely positioned in a stereotaxic frame. Body temperature was kept constant at 37 °C using a homeothermic blanket system (Harvard apparatus, Lewes, DE, USA). A craniectomy of Φ = 4 mm was drilled over the right parietal bone (− 2.5 mm A/P and 2.0 mm lateral from bregma). Brain injury was induced at the craniectomy center using a Φ = 3 mm flat tip connected to an eCCI-6.3 device (Custom Design & Fabrication, LLC, Petersburg, VA, USA) at a velocity of 5.0 m/s, with a 250 ms impact duration and a depth of 2.5 mm. Two severely injured mice (2.5 mm depth) died unexpectedly during the study and were excluded. Kwik-Sil (WPI, Sarasota, FL, USA) was applied over the craniectomy site, and the incision was closed with 4.0 PDO sutures (AD surgical, Sunnyvale, CA, USA).

### EEG implantation

Electrodes were surgically placed into the subjects at 60 days following CCI injury, baed on our well-established protocol^69^, and were left in place for 60 days during EEG monitoring. We utilized a stereotaxic micromotor drill (Stoelting) with a 0.7 mm carbon steel burr drill bit (FST) to create two openings in the skull for the placement of reference electrodes at coordinates (1.00 ML, 1.00 AP) and (− 1.00 ML, − 1.00 AP), along with a ground electrode at (− 1.00 ML, − 5.00 AP). Additionally, we partially drilled two more holes through the skull and secured screws as anchor points. Within 0.5 mm from the dura's surface, we implanted a platinum–iridium electrode with a diameter of 0.125 mm, coated in Teflon, obtained from Plastics One in Roanoke, VA, USA. To secure the electrode in place, we applied dental cement (Stoelting, Wooddale, IL, USA). Sham animals underwent craniectomy surgery and electrode implantation. The electrodes were connected to a commutator (Plastics One) using EEG cables (Plastics One) and subsequently linked to an amplifier (EEG100C, BioPac). The amplifier was set with a gain of 5000, a 100 Hz low-pass filter, a 0.5 Hz high-pass filter, and a 500 Hz sampling rate. BioPac's AcqKnowledge software, version 4.0, was employed for continuous EEG data recording over the course of two months. The identification and incidence of seizure development was reported previously^13^.

### Brain Tissue Preparation for Astrocyte Isolation and RNA Extraction

Isolation of cortical and hippocampal astrocytes was undertaken according to previous methods^70–72^. In brief, at 4 months post-injury, the animals had their electrodes removed, and the cortices and hippocampi from both the 15 CCI-injured mice (2.5 mm depth) and 5 shams were microdissected and separated in ice-cold ACSF (120 mM NaCl, 3.0 mM KCl, 2 mM MgCl, 0.2 mM CaCl, 26.2 mM NaHCO3, 11.1 mM glucose, 5.0 mM HEPES, 3 mM AP5, 3 mM CNQX) that was bubbled with 95% oxygen. The tissue was minced, subjected to dissociation for 15 to 30 min using the Worthington Papain Dissociation Kit, and subsequently triturated and filtered through a 70 µM filter until a single-cell suspension was obtained. Astrocytes were isolated utilizing Miltenyi Biotec’s ACSA-2+ MicroBead kit and then placed on RNAlater (Thermo Fisher Scientific).

### RNA Extraction and Sequencing Analysis

RNA isolation was executed on isolated astrocytes from the brain^70,71^. Subsequently, RNA sequencing was carried out by MedGenome (Foster City, California). Libraries were assembled via the Takara SMART-Seq V4 ultralow-input RNA kit. Sequencing was accomplished using a NovaSeq instrument (Illumina, San Diego CA, USA). Paired-end reads of 2 × 100 bp sequencing runs were generated, with an average of 55 million reads per sample. Trim Galore (v0.6.4) was used to trim bases with quality scores below 30, as well as adapters, from the raw sequencing reads. After this trimming step, only reads exceeding 30 bp in length were mapped to the mm10 reference genome using RSEM (v1.2.28) with bowtie2 (v2.4.1), achieving an average mapping efficiency of 84.2%. For the identification of differentially expressed genes, raw counts were employed using DESeq2 (v1.36.0). Specifically, genes meeting the criteria of having an average TPM greater than 5 in at least one group, a *p* value less than 0.05, and at least a 1.2-fold change were considered as differentially expressed genes. To gain insights into the functional significance of these genes, GO enrichment analysis was conducted using the R package clusterProfiler (v4.4.4) along with org.Mm.eg.db (v3.15.0). The top 10 most significant biological process (BP) terms were selected to generate GO circle plots with the R package GOplot (v1.0.2). GEO accession: #GSE248371. Additionally, an Ingenuity Pathway Analysis (IPA) was performed using Qiagen IPA (v22.0.2.) to further explore the biological pathways and networks related to the differentially expressed genes. All RNA extraction and sequencing analyses were performed on the same mice that received 24/7 EEG evaluation. Included in the analysis were four PTE+ contralateral samples, nine PTE− contralateral samples, four PTE+ ipsilateral samples, and ten PTE− ipsilateral samples. One PTE+ animal was excluded due to a low correlation value, and one PTE− animal was excluded due to a lack of sequencing data.

### Brain tissue preparation, sectioning and staining

Tissue handling procedures were carried out following established protocols as previously outlined^73^. In brief, the mice were humanely euthanized using isoflurane, followed by transcardial perfusion with 1X PBS, and subsequently with 4% paraformaldehyde (PFA). The brains were then immersed in 4% PFA at 4 °C overnight. After this fixation step, the brains underwent cryopreservation and were embedded in an optimal cutting temperature compound (OCT; Fisher Scientific, Waltham, MA, USA), after which they were stored at − 80 °C. Using a CryoStar Cryostat NX70 instrument (Thermo Fisher Scientific, Highpoint, NS, USA), five consecutive coronal sections, each measuring 30 μm in thickness and spaced 450 μm apart, were prepared and mounted on pre-coated, charged slides. Coronal serial sections were initially fixed using 10% buffered formalin, followed by three washes in 1 × PBS. Subsequently, they were blocked with a solution consisting of 2% cold-water fish gelatin (Sigma Aldrich, Inc., St. Louis, MO, USA) and 0.2% Triton. These sections were then subjected to an overnight incubation at 4 °C in the block buffer, along with primary antibodies. The primary antibodies used included Anti-Cst3 (R&D, # AF1238-SP), and Anti-GFAP (Cell Signaling, #12380). Following the antibody incubation, the slides underwent a series of PBS washes (3 times) before being exposed to secondary antibodies. Finally, the slides were mounted in a medium containing DAPI (SouthernBiotech, Birmingham, AL, USA). Images were captured using a Nikon ECLIPSE Ti2 inverted confocal microscope equipped with a motorized stage and a Nikon C2 laser system.

### Supplementary Information

Supplementary Information.

## Data Availability

The datasets generated during the current study are available in the NCBI GEO repository, GEO accession: #GSE248371. For reviewer access: kpcpcsqipnyjrux.

## Abbreviations

PTE: Post-traumatic epilepsy
TBI: Traumatic brain injury
CCI: Controlled cortical impact
DEG: Differentially expressed genes
