# Supplementary material for: Transcriptomic alterations in cortical astrocytes following the development of post-traumatic epilepsy
Source: Sci Rep. 2024 Apr 10;14:8367. doi: 10.1038/s41598-024-58904-z (PMC11006850; doi:10.1038/s41598-024-58904-z)
Supplement: Supplementary file 1 — Supplementary Information. [file 41598_2024_58904_MOESM1_ESM.docx]

**
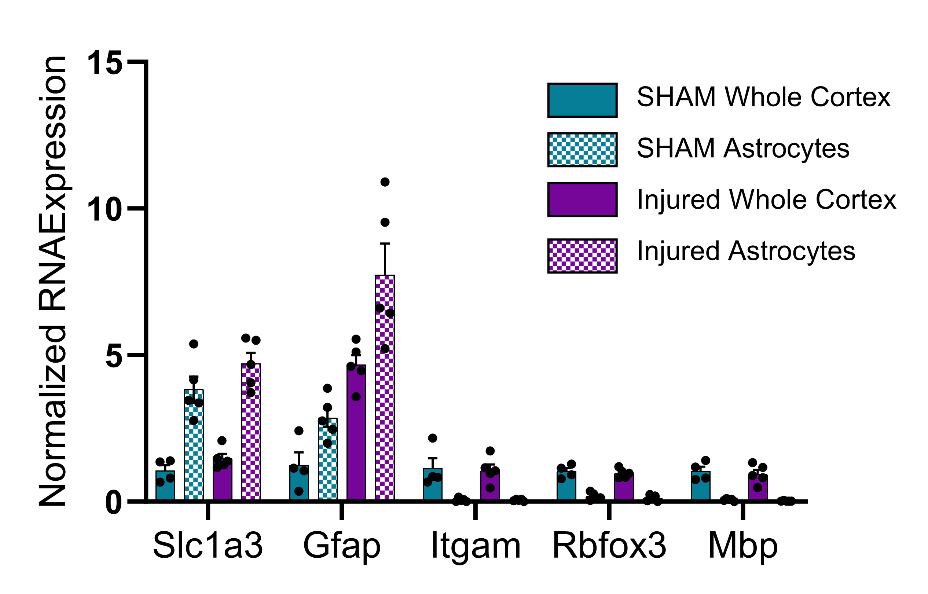
**

**Supplemental Figure 1. Enrichment of astrocyte-specific gene in isolated astrocytes from cortex at 4 months post-injury.** qPCR was performed on astrocytes isolated from the injured cortex and compared to whole cortex showing enrichment. Astrocytes show higher transcript levels of *Slc1a3, Gfap* and reduced *Itgam*, *Rbfox3* and *Mbp*. Genes were compared to *Gapdh* and normalized to sham whole cortex.
